# Shoot and Root Traits Underlying Genotypic Variation in Early Vigor and Nutrient Accumulation in Spring Wheat Grown in High-Latitude Light Conditions

**DOI:** 10.3390/plants10010174

**Published:** 2021-01-18

**Authors:** Hui Liu, Fabio Fiorani, Ortrud Jäck, Tino Colombi, Kerstin A. Nagel, Martin Weih

**Affiliations:** 1 Department of Crop Production Ecology, Swedish University of Agricultural Sciences, 75007 Uppsala, Sweden; ortrud.jack@slu.se (O.J.); martin.weih@slu.se (M.W.); 2 Institute for Bio and Geosciences-2, Plant Sciences, Forschungszentrum Jülich GmbH, 52425 Jülich, Germany; f.fiorani@fz-juelich.de (F.F.); k.nagel@fz-juelich.de (K.A.N.); 3 Department of Soil and Environment, Swedish University of Agricultural Sciences, 75007 Uppsala, Sweden; tino.colombi@slu.se

**Keywords:** early vigor, high-latitudes, leaf area, nutrient, root growth, wheat

## Abstract

Plants with improved nutrient use efficiency are needed to maintain and enhance future crop plant production. The aim of this study was to explore candidate traits for pre-breeding to improve nutrient accumulation and early vigor of spring wheat grown at high latitudes. We quantified shoot and root traits together with nutrient accumulation in nine contrasting spring wheat genotypes grown in rhizoboxes for 20 days in a greenhouse. Whole-plant relative growth rate was here correlated with leaf area productivity and plant nitrogen productivity, but not leaf area ratio. Furthermore, the total leaf area was correlated with the accumulation of six macronutrients, and could be suggested as a candidate trait for the pre-breeding towards improved nutrient accumulation and early vigor in wheat to be grown in high-latitude environments. Depending on the nutrient of interest, different root system traits were identified as relevant for their accumulation. Accumulation of nitrogen, potassium, sulfur and calcium was correlated with lateral root length, whilst accumulation of phosphorus and magnesium was correlated with main root length. Therefore, special attention needs to be paid to specific root system traits in the breeding of wheat towards improved nutrient accumulation to counteract the suboptimal uptake of some nutrient elements.

## 1. Introduction

Nutrients are among the most critical factors limiting plant growth, and mineral fertilization is widely used to enhance crop yields. However, as the production of mineral fertilizers consumes large amounts of energy, their application is a key cost factor in the farm economy and often associated with environmental issues [1,2,3]. Therefore, breeding new crop genotypes with improved nutrient accumulation and use efficiencies is required for both economic and environmental reasons.

Previous studies pointed out the importance of early vigor, which has been defined previously as the early vigorous growth of shoots and roots [4,5,6], for the acquisition and use of nutrient resources by crops in the early growing season. However, the assessment of early vigor was mostly based on shoot traits rather than root traits [4,5,6,7,8,9,10,11]. Exploiting genotypic variation in root traits is suggested to be a promising approach to improve nutrient accumulation and use efficiency of crops as well as plant performance under drought and nutrient stress [12]. Studies in wheat, maize, and common bean highlighted links between shoot growth and root system traits, such as number and length of main and lateral roots under low nutrient and water availability [13,14,15,16]. Similarly, root anatomical traits such as cortical aerenchyma and cortical cell diameter have been associated with plant tolerance to low nutrient availability or drought [17,18,19]. In contrast to plant nutrients that are highly mobile in soil (e.g., N), the accumulation of less mobile nutrients (e.g., P) is more dependent on root growth than shoot growth [20]. Therefore, a plant that gives lower priority to root growth is more likely to accumulate lower amounts of immobile nutrients rather than mobile nutrients, resulting in suboptimal nutrient ratios (e.g., P-to-N ratio). Due to the strong functional links between above and below ground plant parts, a focus on both root and shoot traits during early growth stages is needed when targeting the growth improvement of spring crops in a high-latitude cool climate with long diurnal photoperiods and short growing seasons. Spring wheat is one of the most important spring crops that can be cultivated in high-latitude regions, which we here refer to the regions north of 55° N, e.g., Sweden, Norway, Denmark, and Canada. The need for spring wheat breeding programs especially for the environmental conditions at high-latitudes has been highlighted, and spring wheat breeders show an interest in the potential genetic variation in early vigor-related root traits and their association with efficient nutrient accumulation and use [21]. Roots are challenging to investigate, but non-invasive, high-throughput phenotyping methods are available for identifying genotype-specific root traits especially under controlled growth conditions [22,23,24,25].

Functional plant growth analysis is used to analyze the accumulation of whole plant biomass and its allocation among different plant organs in relation to the acquisition of above ground and below ground resources [26]. In this context, the calculation of relative growth rate (RGR, the increase in whole plant biomass per unit of initial biomass and time) and the separation of different components driving RGR are central elements, which mechanistically link plant growth to growth-limiting resources such as light and nutrients. The evaluation of the potential drivers for variation in RGR is context-dependent. When light is expected to be a key factor for growth, RGR is separated into the components including leaf area ratio (LAR, leaf area per unit of whole plant biomass) and leaf area productivity (LAP, whole plant biomass production per unit of leaf area and time, also called net assimilation rate or unit leaf rate; [27,28]). When N is expected to be a key factor for growth, RGR is separated into the components including mean plant N concentration and N productivity (whole plant biomass production per unit of plant N pool and time; [27,28]). Different drivers for explaining variations in RGR may be found in different species and growing conditions [28,29,30,31]. For example, the relative importance of LAP and LAR in determining RGR appears to depend on light environment [31,32]. Furthermore, Shipley [31] identified LAP as the best general predictor of variation in RGR of herbaceous species grown under high-light conditions. In this context, it is interesting that long-day treatments of about 16 h, typical for high-latitude environments, have been shown to promote leaf area and biomass growth in many grass species; and that light supply at a low irradiance over a longer period can be more efficient than a high irradiance short-day treatment [33]. It is therefore possible that the importance of LAP for variation in RGR previously observed under high-light conditions is also seen in spring wheat grown under the long diurnal photoperiod typical at sowing time at high-latitudes.

In wheat breeding, the efforts to improve nutrient uptake have so far mainly focused on N, whilst other nutrients were poorly considered [34,35]. It is possible that the strong focus on N alone has jeopardized greater progress in breeding towards improved nutrient accumulation and use, because plant growth in some developmental stages may be co-limited by more than a single nutrient [36,37]. At least 16 nutrient elements are known to be essential for all higher plants [38]. They can be classified into macronutrients and micronutrients according to their structural, enzymatic, energetic and osmotic functionalities [36]. Recent investigations in wheat field-grown in Sweden showed that the concentrations of several macronutrient elements were positively correlated to the concentration of N, whereas micronutrients were usually not accumulated in proportion to N [39,40]. Nutrient accumulation in plants can be evaluated in relation to optimum nutrition by considering optimum N-based element ratios (e.g., P-to-N ratio) for plant growth, which have been reported by Knecht and Göransson [41] for several nutrient elements and for different functional groups of plants. Thus, according to Weih et al. [37], observed N-based element ratios can be compared with the corresponding expected N-based element ratios derived from the literature. The proportional acquisition of nutrients in relation to each other can also be evaluated using products of element concentrations and the calculation of scaling exponents. This allows to explore for example if the concentrations of the other elements increase faster or slower than the concentrations of N and P [42].

The overall aim of this study was to explore candidate shoot and root traits that can be used in breeding to improve nutrient accumulation and early plant vigor in spring wheat under high-latitude conditions. The specific aims were: (1) to investigate the main driving variables for the variation of relative growth rate in juvenile spring wheat plants; (2) to identify shoot and root traits associated with the accumulations of N, P, K, S, Ca, and Mg; and (3) to study the relationships between the accumulations of different nutrients, and to evaluate them with the approaches of the optimum N-based element ratios and the stoichiometric niche volumes. A greenhouse experiment was conducted with nine contrasting spring wheat genotypes grown for 20 days in rhizoboxes and assessed in an automated phenotyping system. Functional growth analysis was performed on juvenile spring wheat plants to assess mechanistic links between plant traits important for biomass and nutrient accumulation. We expected the genotypes to vary in growth and the underlying traits, and explored the following hypotheses: (H1) under the long-photoperiod conditions typical at high-latitudes, leaf area productivity and plant N productivity are reliable predictors for variation in relative growth rate of whole plant biomass at early growth stage; (H2) the accumulations of macronutrients are positively correlated with leaf area, as well as root morphological and anatomical traits such as root number and length, root system depth and width, cortical aerenchyma and cell diameter; (H3) the macronutrients P, K, S, Ca, and Mg accumulated in proportion to N across all genotypes.

## 2. Results

### 2.1. There Is Considerable Trait Variation across the Nine Genotypes

**Figure 1:**
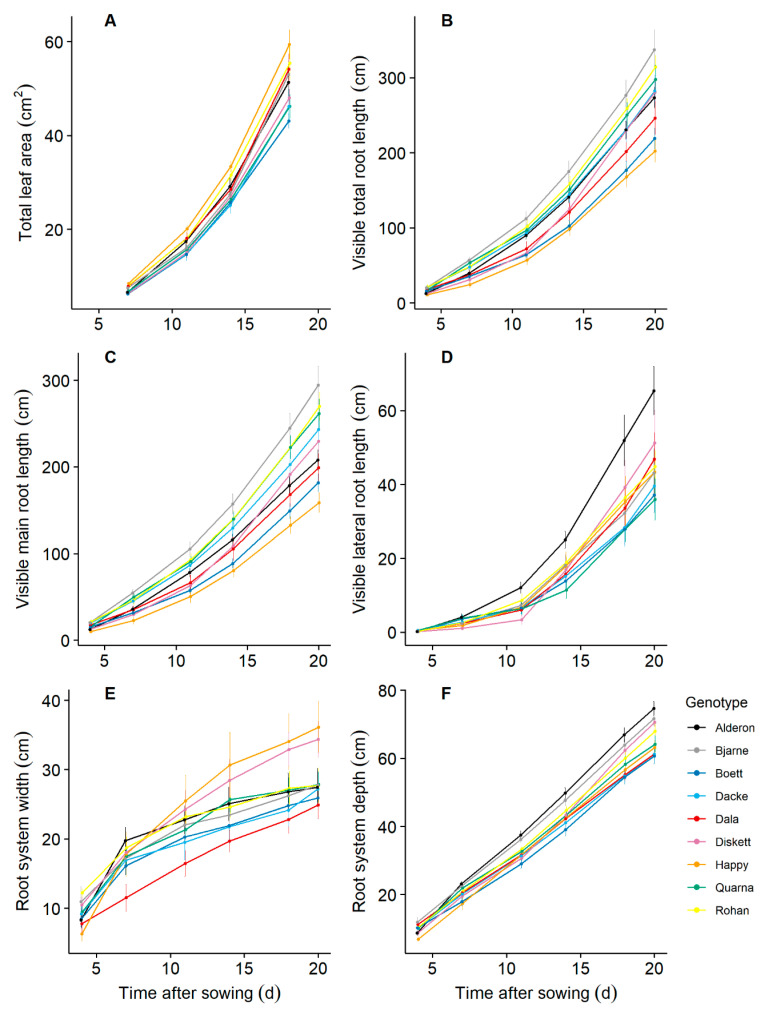
Leaf and root development dynamics along 20 days of growth for nine spring wheat genotypes grown under controlled conditions in a phenotyping facility. The total leaf area was estimated from non-destructive measurements and calculated as leaf length × maximum width × 0.858 [43]. The root parameters were quantified using root images from the automated phenotyping system. Each value represents the mean ± standard error (n = 8).

Increase over time of total leaf area, visible total root length, visible main root length, visible lateral root length as well as root system width and depth varied significantly among the nine genotypes (Figure 1), which were reflected by significant time by genotype interactions in repeated measures ANOVA (Supplementary Table S1). For example, the genotype ‘Alderon’ grew the longest lateral roots and deepest root system consistently throughout the entire growth period; whilst ‘Diskett’ and ‘Happy’ had slower root system width growth than the other genotypes in the first seven days, but considerably faster root system width growth thereafter.

After 20 days of growth, significant differences among genotypes were found for most of the phenotypic traits analyzed (Table 1), including all root traits extracted from non-destructive image analysis (i.e., visible total root length, visible main root length, visible lateral root length, visible root system width and depth), leaf chlorophyll content (i.e., SPAD leaf1, SPAD leaf3), and many shoot and root traits from the destructive sampling (e.g., shoot biomass, root biomass, total leaf area, total root length, lateral root length). We found significant genotype effects on cross-sectional root area, cortex area and stele area of the nodal roots, indicating that nodal root diameter differed among genotypes. Other root anatomical traits such as cortical aerenchyma and cortical cell diameter did not differ significantly among genotypes (Table 1).

### 2.2. Leaf Area Productivity and Plant Nitrogen Productivity Drive the Variation in Relative Growth Rate of Whole Plant Biomass among Genotypes

**Figure 2:**
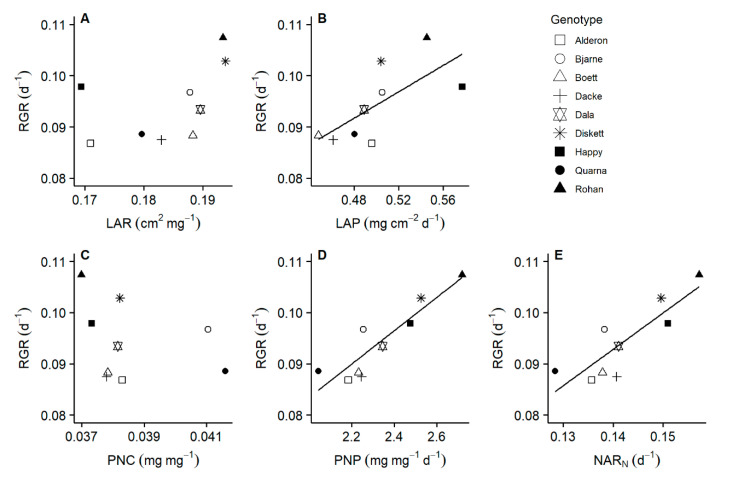
Relative growth rate of whole plant biomass and its driving variables for nine spring wheat genotypes. Relative growth rate (RGR) was calculated using the whole plant biomasses at days 1 and 20. Leaf area ratio (LAR) and leaf area productivity (LAP) were calculated using the total leaf areas at days 7 and 20. Plant N concentration (PNC), plant N productivity (PNP) and relative N accumulation rate (NAR_N_) were calculated using the N concentrations at days 1 and 20. Linear regressions: (**A**) r^2^ = 0.135, *p* = 0.178; (**B**) r^2^ = 0.416, *p* = 0.036; (**C**) r^2^ =−0.049, *p* = 0.456; (**D**) r^2^ = 0.797, *p* < 0.001; (**E**) r^2^ = 0.690, *p* = 0.003.

Relative growth rate of whole plant biomass (sum of root and shoot biomass) ranged from 0.09 to 0.11 d^−1^ across the investigated nine genotypes. Leaf area productivity was a better predictor of the variation in relative growth rate of whole plant biomass than leaf area ratio across all genotypes (Figure 2A,B). In terms of plant N use, increased relative growth rate of whole plant biomass was more related to higher plant N productivity than N concentration (Figure 2C,D). The relative growth rate of whole plant biomass was a linear function of the relative N accumulation rate (Figure 2). Total leaf area (from destructive sampling) was positively correlated with both relative growth rate of whole plant biomass and leaf area productivity. Nodal root number was positively correlated with both relative growth rate of whole plant biomass and plant N productivity (Supplementary Table S2).

**Figure 3:**
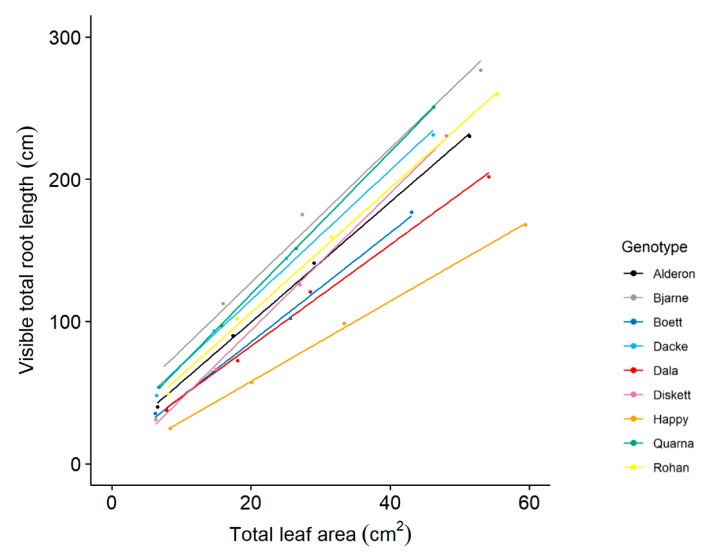
Allocation between leaf and root for nine spring wheat genotypes grown under controlled conditions in a phenotyping facility. The four values for each genotype represent the means of eight replicates from days 7, 11, 14 and 18, respectively. Linear regressions: ‘Alderon’, r^2^ = 0.998, *p* < 0.001; ‘Bjarne’, r^2^ = 0.980, *p* = 0.007; ‘Boett’, r^2^ = 0.995, *p* = 0.002; ‘Dacke’, r^2^ = 0.993, *p* = 0.002; ‘Dala’, r^2^ = 0.992, *p* = 0.003; ‘Diskett’, r^2^ = 0.998, *p* < 0.001; ‘Happy’, r^2^ = 0.999, *p* < 0.001; ‘Quarna’, r^2^ = 0.999, *p* < 0.001; ‘Rohan’, r^2^ = 0.997, *p* = 0.001.

Visible total root length (assessed non-destructively) was plotted against total leaf area (assessed non-destructively) to show genotypic difference in the allocation between root and leaf, with the data from days 7, 11, 14, and 18 (Figure 3). Visible total root length consistently increased with total leaf area, but the slopes of the relationships varied significantly among the genotypes (*p* < 0.001). For example, the genotype ‘Happy’ had the smallest slope, which indicates that its leaf growth was prioritized over root growth in comparison with the other genotypes.

### 2.3. Main and Lateral Root Length Are Associated with Accumulation of Different Nutrients

**Figure 4:**
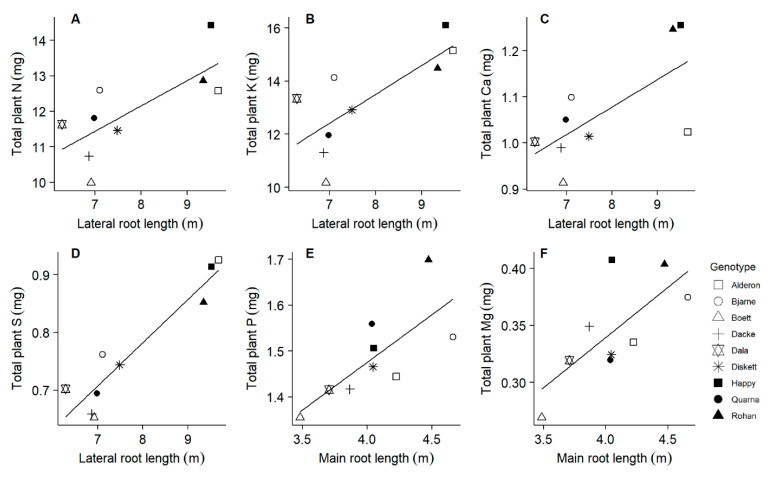
Relationships between root length and the pools of macronutrients in whole plants at day 20. Root length was measured destructively with a scanner at day 20. Linear regressions: (**A**) r^2^ = 0.532, *p* = 0.026; (**B**) r^2^ = 0.573, *p* = 0.018; (**C**) r^2^ = 0.460, *p* = 0.045; (**D**) r^2^ = 0.887, *p* < 0.001; (**E**) r^2^ = 0.558, *p* = 0.021; (**F**) r^2^ = 0.516, *p* = 0.029.

**Figure 5:**
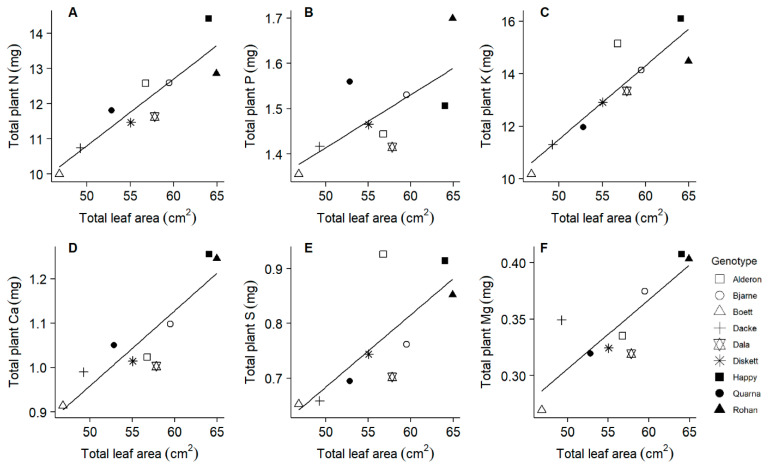
Relationships between total leaf area and the pools of macronutrients in whole plants at day 20. Total leaf area was measured destructively with an area meter at day 20. Linear regressions: (**A**) r^2^ = 0.810, *p* < 0.001; (**B**) r^2^ = 0.498, *p* = 0.034; (**C**) r^2^ = 0.814, *p* < 0.001; (**D**) r^2^ = 0.812, *p* < 0.001; (**E**) r^2^ = 0.586, *p* = 0.016; (**F**) r^2^ = 0.718, *p* = 0.004.

After 20 days of growth, lateral root length (from destructive sampling) ranged from 6.29 to 9.67 m, whilst main root length (from destructive sampling) ranged from 3.48 to 4.66 m. Across all genotypes, lateral root length was positively correlated with the accumulation of N, K, Ca, and S, but not with P or Mg (Table 2). The genotypes ‘Rohan’, ‘Happy’ and ‘Alderon’, with longer lateral root lengths, accumulated larger amounts of N, K and S in comparison with the other genotypes (Figure 4). Main root length was positively correlated with the accumulation of P and Mg, but not with N, K, Ca, or S (Table 2). The genotype ‘Rohan’, with particularly long main roots, accumulated high amounts of P and Mg (Figure 4). Total leaf area (from destructive sampling) ranged from 46.8 to 64.9 cm^2^, and it was positively correlated with the accumulation of all the six macronutrients (Table 2). The genotypes ‘Rohan’ and ‘Happy’, with large leaf areas, also accumulated high amounts of N, P, K, Ca, S, and Mg (Figure 5). Shoot biomass was correlated with the accumulation of N, K, Ca, S, and Mg, but not P (Table 2).

Nutrient accumulation was not correlated with the root anatomical traits (Table 2) and in most cases, the non-destructively assessed root traits (i.e., visible total root length, visible main root length, visible lateral root length, visible root system width and depth). The only exceptions were visible root system width at day 11, which was correlated with S accumulation; and visible lateral root length at day 14, which was correlated with S and K accumulation (Supplementary Table S3).

### 2.4. All Macronutrients Except Phosphorus Scale Similar to Nitrogen

**Figure 6:**
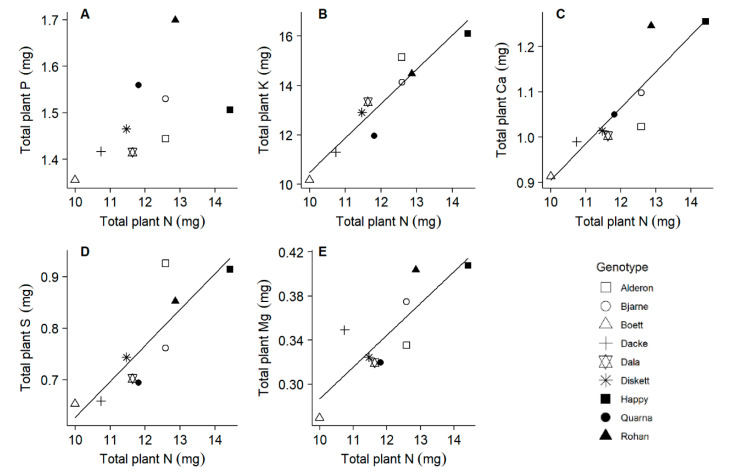
Relationships between the pools of various macronutrients in whole plants at day 20. Linear regressions: (**A**) r^2^ = 0.241, *p* = 0.102; (**B**) r^2^ = 0.876, *p* < 0.001; (**C**) r^2^ = 0.761, *p* = 0.001; (**D**) r^2^ = 0.697, *p* = 0.003; (**E**) r^2^ = 0.662, *p* = 0.005.

The accumulated N ranged from 9.99 to 14.42 mg plant^−1^, whilst P ranged from 1.36 to 1.70 mg plant^−1^ in the nine genotypes. Across all genotypes, the accumulations of K, Ca, S and Mg were linear functions of the accumulation of N, while P accumulation scaled differently to N accumulation (Figure 6). No significant correlation was observed between the accumulation of N and any of the micronutrients (Fe, Mn, Zn, Cu; Supplementary Table S4). We found a significant positive correlation between the two different estimates of N productivity (Pearson r = 0.80, *p* = 0.010, n = 9). When using the inverses of the plant N and P concentrations at final harvest as surrogates to estimate N and P productivity, the whole plant biomass was a function of P productivity modified by the N productivity (ln (whole plant biomass) = 0.97 × ln (P productivity) − 2.96 × ln (N productivity) − 4.60, r^2^ = 0.70, *p* = 0.005; Supplementary Figure S1).

### 2.5. Suboptimal P-to-N and Mg-to-N Ratios Were Observed in Some Genotypes

**Figure 7:**
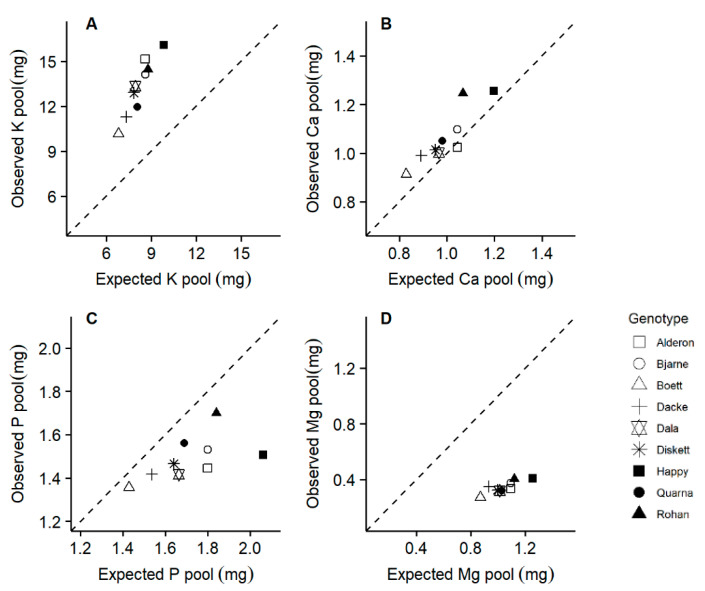
Comparison of the observed nutrient pools at day 20 and the expected pools for achieving optimum nutrient ratios. Expected nutrient pools were calculated based on the suggested optimum N:P:K:Ca:Mg ratios of 100:14.3:68.3:8.3:8.7 by Knecht and Göransson [41]. The broken lines indicate where the observed nutrient pools equal the expected values.

The observed K and Ca pools were higher or similar to the expected pools for achieving optimum nutrient ratios, whilst the observed P and Mg pools were lower than the corresponding expected pools (Figure 7). For example, the genotype ‘Happy’ appears to run a greater risk of suboptimal P accumulation when compared with the other genotypes. In addition, compared to N and P, the concentrations of the other elements (K, Ca, S, Mg, Cu, Fe, Mn and Zn) increased at a slower rate, which was reflected by a negative scaling exponent (−0.881) for the niche volume of N and P as related to the volume of the other nutrient elements (Supplementary Figure S2).

## 3. Discussion

This study was focusing on the early growth stages of spring wheat, because the first three weeks after sowing are critical for nutrient uptake and plant development especially in the cool environmental conditions and short growing seasons at high-latitudes. The results provide a first important step in linking the genetic differences in nutrient accumulation processes at early growth stages, which are important for the growth and development of all later stages, to the growth and yield formation at later growth stages. By exploring trait relationships across nine commercially used genotypes in a controlled growth environment, we found considerable variation in various root and shoot traits related to early vigor, and identified several traits that are likely to control much of the variations in growth and nutrient accumulation observed across these genotypes. We found indications for suboptimal uptake of P especially in a genotype (i.e., ‘Happy’) that prioritized leaf growth over root growth during the early seedling phase. The strengths of this study include the functional growth analysis approach performed on both above and below ground plant parts, facilitating the identification of mechanistic links between traits for some contrasting genotypes; the use of non-invasive methodologies to follow root growth during the critical early phase of seedling development; and the consideration of many nutrients other than N. A limitation of this study is its correlational research design, which cannot be used to draw safe conclusions regarding the causal relationships among the measured variables, for example, between leaf area and nutrient accumulation. However, in an attempt to identify easy-to-measure candidate traits for the selection of plants with high nutrient accumulation, recommendations can be made even without unravelling all causal relationships involved. For example in our study, total leaf area can be suggested as an indicator of improved nutrient accumulation when selecting wheat lines, because measuring leaf area is much cheaper and faster than determining nutrient contents. Our study focused on the early seedling phase investigated in a controlled environment and will be a basis for further studies to translate the results to mature plants grown under field conditions.

### 3.1. Genotypic Variation in Relative Growth Rate of Whole Plant Biomass and Its Determinants

As proposed in our first hypothesis, leaf area productivity (LAP) was identified to be a better predictor of variation in relative growth rate of whole plant biomass (RGR) compared to leaf area ratio (LAR) under the 16 h photoperiods used here. Also in a broad meta-analysis including 334 herbaceous species [31], LAP was found to be a better driver for variation in RGR than the two LAR components (specific leaf area and leaf mass ratio) especially under high-light conditions. Thus, the relative importance of LAP and LAR in determining RGR has been considered to depend on the light conditions, with the importance of LAP increasing with irradiance [31]. Still, in a study on 24 herbaceous species grown under slightly higher irradiance but shorter days (day length of 14 h and average quantum flux density of 315 µmol m^−2^ s^−1^) than our plants experienced (day length of 16 h and average quantum flux density of 144 µmol m^−2^ s^−1^), RGR was associated with LAR but not LAP [30]. It has previously been shown that a low irradiance treatment given over a longer period can be more efficient in supporting plant growth than a high irradiance short-day treatment [33]; and that biomass production of a high-latitude grass species increased linearly with photoperiod between 10 and 16 h [44]. Our results therefore indicate that the importance of LAP for variation in RGR previously observed under high-light conditions, is also relevant in spring wheat grown under the relatively long photoperiod typical at sowing time at high-latitudes such as Northern Europe. The photoperiod, instead of irradiance, could thus be an equally important factor for switching the relative importance of LAP and LAR in determining RGR. Because we found a strong and significant relationship between total leaf area and LAP, our results also indicate that greater partitioning to leaf area growth (rather than leaf thickening) resulted in enhanced light interception leading to greater LAP in these juvenile plants. Leaf area can be assessed with optical methods well suited for high-throughput screening of plants [45], and we have here demonstrated that leaf area can be used as an indicator for LAP and RGR during the juvenile (purely vegetative) growth of spring wheat in a long-day environment.

Because leaves are the most important sinks for plant N, the enhanced light interception with greater leaf area observed here is probably also the reason for the significant relationship found between RGR and plant N productivity. Thus, among the genotypes and conditions investigated here, RGR was driven more by the allocation of N to leaf area, which determines the productivity per unit N, than the mass-based concentration of N in the plant [27]. Nitrogen was however possibly not the only growth-limiting nutrient for all genotypes in this study, because we found indications for suboptimal P-to-N ratios for certain genotypes (Figure 7). Thus, 70% of the variation in the final (20 days of growth) whole plant biomass was indeed predicted by a surrogate of P productivity (calculated as the inverse of P concentration) modified by N productivity (Supplementary Figure S1). Phosphorus is strongly involved in various key photosynthetic processes [46] and associated with carbon assimilation and productivity [47], which has also been shown for cereals grown in the field [48]. For some of the genotypes investigated here, leaf P could thus have limited LAP more than leaf N at least during the vegetative seedling phase evaluated here.

### 3.2. Association of Shoot and Root Traits with the Accumulation of Individual Nutrient Elements

Leaf area as a main driver of growth requires sufficient allocation of nutrients. We therefore expected the nutrient pools accumulated by the nine genotypes to be correlated to their leaf areas or shoot biomasses. Evidence for this expectation comes also from other studies reporting strong correlations between N uptake and shoot biomass [4,6,10]. In line with our hypothesis, leaf area was positively correlated with the accumulations of the nutrients important for photosynthesis, i.e., N, P, K, Ca, S, and Mg (Figure 5). On the one hand, the greater transpiration flow caused by larger leaf area could also have resulted in enhanced nutrient uptake rate [49]; assuming mostly passive nutrient uptake processes which are likely to dominate for the uptake of K, Ca, and Mg but not of N, P, and S for which active processes located in roots are important [50]. On the other hand, the genotypes with greater total leaf areas had also longer roots (Table 2), and the accumulations of several nutrient elements were correlated also with different root traits (Figure 4). In addition, some genotypes (e.g., ‘Happy’) progressively grew shorter roots in proportion to leaf area when compared to other genotypes (Figure 3). Because we found especially P and Mg accumulation to be lower in those genotypes with shorter main root length, it is possible that the smaller slope of the relationships between total leaf area and total root length(Figure 3) of ‘Happy’ is linked to the suboptimal P and Mg pools in this genotype (Figure 7).

It was shown that maize genotypes with long lateral roots have higher N accumulation than genotypes with rather short lateral roots [16]. In agreement with these results, we found a positive correlation between lateral root length and N accumulation in the investigated spring wheat genotypes. Moreover, longer lateral roots also improved K, Ca, and S accumulation (Figure 4). Lateral roots have also been shown to contribute to P acquisition, especially under low P availability conditions [1,51]. In contrast to these results, we found P accumulation to increase with longer main root length (Figure 4) instead of lateral root length. This discrepancy in results could possibly be attributed to the frequent and sufficient P supply during our experiment, which does not adequately reflect the conditions in the field. In addition, Mg accumulation increased with longer main root length in our study (Figure 4). Our results suggest that different root traits need probably to be targeted in plant breeding to improve the acquisition of specific nutrient elements.

Root diameter differed among genotypes as indicated by significant genotype effects on cross-sectional root area, cortex area and stele area, which corresponds to previous studies in wheat [52,53]. In contrast, root cortical aerenchyma and cortical cell diameter did not significantly differ among the genotypes (Table 1). It has been shown that genotypic variation in these traits is more pronounced when plants are exposed to edaphic stress [19,53]. Similarly, effects of cortical aerenchyma on nutrient acquisition were observed to be particularly relevant under low nutrient availability [19]. Our study did not include any stress treatment, which probably explains the lack of genotypic differences in root cortical aerenchyma and cortical cell diameter, and the lack of relationships between these traits and nutrient accumulation.

### 3.3. Nutrient Accumulation and Stoichiometry, and Optimum Nutrient Ratios

To our knowledge, this study is first in analyzing various nutrient elements in young wheat seedlings. We had to use pooled plant samples to allow for reliable element determinations, thus not allowing statistical evaluation of the variation between replicates within genotypes. Similar to the element concentration patterns investigated in field-grown winter wheat at stem elongation and flowering [39,54], the accumulation of all macronutrients except P also scaled similar to N in our juvenile seedlings, partly supporting our third hypothesis. Some genotypes, notably ‘Quarna’ and ‘Rohan’, increased their P pools strongly in proportion to N (strong P accumulators), whilst other genotypes, e.g., ‘Happy’ and ‘Alderon’, showed much weaker P accumulation in proportion to N (weak P accumulators) (Figure 6A). This indicates that there is genetic variation in the proportion of P to N accumulation across the plant material investigated here; and the negative scaling exponent for the niche volumes of N and P vs. the other nutrients [42] indicates that there is genetic variation also with respect to the proportional accumulation of other elements. It is interesting that the genotype ‘Happy’, with a weak P accumulation in proportion to N, and consequently the strongest deviation from an optimal P-to-N ratio (Figure 7C), was also the one with the smallest slope in the relationship between root and leaf development (Figure 3). Thus, the non-destructive quantification of both root length and leaf area, which can be done in automated phenotyping facilities, could possibly be used for characterizing the proportional P uptake in relation to N. Furthermore, investigations are needed to evaluate whether the suboptimal P accumulation, as apparent in some of our genotypes after 20 days of growth at tillering stage, continues towards anthesis and thereafter and is associated with lower grain yield.

The observed P and Mg pools were lower than the expected pools achieving optimum nutrient ratios in some genotypes (e.g., ‘Happy’; Figure 7), a pattern observed also in some field-grown winter wheat especially at high fertilization levels [37]. Especially for Mg, the reported optimum nutrient ratios, and thus the expected nutrient pools in our study, may not be valid for all plants and growth conditions, because they may depend on the plant growth rate or nutrient supply rate [41]. Nevertheless, suboptimal Mg contents have been highlighted as a potential issue in many crops [55], and should be further investigated also with respect to possible genotypic variation.

### 3.4. Implications for Breeding

The focus of this paper is on early vigor-related traits, which are considered important especially for spring crops grown under short growing seasons at high-latitude regions. Early vigor-related growth traits are frequently regarded challenging for phenotyping, because currently available phenotyping approaches for these traits still rely on manual scoring which is time-consuming on large breeding populations [21]. Based on these difficulties, there are only few studies on the links between different early vigor-related traits and their relationships to nutrient accumulation and use in wheat, and only few genotypes have been examined [4,5,6,9,10,56]. We found considerable genotypic variation in early vigor-related root and shoot traits among the nine spring wheat genotypes used in this study. Under the relatively long photoperiods (16 h) in our investigation, the genotypic variation in relative growth rate of whole plant biomass was linked to the variations in leaf area productivity and plant N productivity, and leaf area productivity was highly correlated with the total leaf area. Also, the accumulation of the various nutrients investigated here varied among the genotypes, and this variation was reflected by the variations in both leaf area and root system traits. Our study demonstrates that breeders may need to select for specific root traits to improve the accumulation of different nutrient elements. Our study could provide useful information for breeding of crops with improved nutrient accumulation grown under well-fertilized conditions (typical for many agricultural fields), which has rarely been reported [57]. Since root systems often show plastic responses to nutrient availability [12,20,25,34,43], the improvement of nutrient accumulation of crops grown in the nutrient poor agriculture systems may need different breeding strategies.

Not all macronutrients were accumulated proportionally in relation to N, noteworthy exception was P. We found genotypic variation in the proportion of P to N accumulation across the plant material investigated here, which possibly could be monitored non-destructively by assessing root length and leaf area in automated phenotyping facilities. Consideration of P-to-N stoichiometry, and the possibility of P limitation during some growth stages, in plant breeding is probably important, because there are indications of suboptimal P supply in field-grown wheat [37] and other cereals, and feasible tools are available to rapidly detect P limitation of photosynthesis also in the field [48]. In this context, it is interesting that low P concentration in seeds has been suggested as a promising trait to improve P use efficiency, although low seed P concentration may be associated with reduced seedling vigor [58,59]. Considering Mg and our observations of possibly suboptimal Mg-to-N ratios, further research is needed to follow Mg stoichiometry during crop growth, because the possibility of Mg deficiencies in crops has been highlighted [55,60].

## 4. Materials and Methods

### 4.1. Plant Material and Experimental Setup

Nine spring wheat genotypes ‘Bjarne’, ‘Boett’, ‘Dacke’, ‘Diskett’, ‘Happy’, ‘KWS Alderon’ (Alderon), ‘Quarna’, ‘Rohan’ and a landrace originated from in Dalecarlia (Dala) were used for the study (Supplementary Table S5). They were grown in the automated *GrowScreen-Rhizo 1* phenotyping platform [23] for 20 days in November 2017. The experimental layout was a randomized complete block design with eight replicate blocks and nine genotypes. One plant was grown in each rhizobox (90 × 70 × 5 cm). Overall, 72 rhizoboxes were used.

The rhizoboxes were filled with sieved peat substrate (Nullerde Einheitserde; Balster Einheitserdewerk, Fröndenberg, Germany), which had a pH of 6.4 and was characterized by low nutrient concentration (total N concentration of 4 mg/g, ammonium acetate-lactate extractable P, K, Ca, Mg concentrations of 0.06, 0.14, 0.85, 0.46 mg/g, respectively). Before sowing, the mean seed fresh weight was determined by weighing 1000 seeds per genotype. Seeds of uniform size were selected and pre-germinated on wet filter paper at 20 °C for two days in the dark. After germination, seedlings of uniform growth (seminal root length of 2–3 cm) were transplanted (sowing depth of 2 cm) into rhizoboxes. All plants were irrigated twice per day with 100 mL of deionized water and supplied three times per week with 200 mL of 100% modified Hoagland solution (5 mM KNO_3_, 5 mM Ca(NO_3_)_2_, 2 mM MgSO_4_, 1 mM KH_2_PO_4_, plus trace elements; [61]). Climate conditions in the greenhouse were monitored during the experiment. Day light was set to 16 h to represent typical photoperiods for the time of spring wheat sowing in high-latitude regions. Supplementary illumination (SON-T AGRO 400, Philips, Amsterdam, The Netherlands) was automatically turned on when the ambient light intensity outside the greenhouse was <400 µmol m^−2^ s^−1^ between 6:00 and 22:00. The mean photosynthetic photon flux density was 144 µmol m^−2^ s^−1^ at plant level during the 16 h. Day/night mean temperatures were 24/19 °C, and the relative humidity was between 30 and 72% (Supplementary Figure S3).

### 4.2. Quantification of Phenotypic Traits

Non-destructive measurements: Root system traits (i.e., visible total root length, visible main root length, visible lateral root length, visible root system width and depth) were quantified non-destructively twice per week during the experiment, using the automated *GrowScreen-Rhizo 1* phenotyping system and the image-based software tool *GROWSCREEN-Root* [23]. Six time points (days 4, 7, 11, 14, 18, and 20 after sowing) were used to perform the root measurements and create root developmental dynamics curves. Four time points (days 7, 11, 14, and 18) were used to non-destructively perform leaf area measurements and create leaf developmental dynamics curves. The length and maximum width of each leaf were measured manually with a ruler twice per week. The leaf areas were calculated according to the following equation:

Leaf area = leaf length × maximum width × *k* (1)

where *k* is the shape factor, which is 0.858 for wheat leaves [43]. The total leaf area of each plant was calculated as the sum of all the leaves. At final harvest (day 20), leaf greenness based on SPAD units (SPAD-502Plus, Konica Minolta, Japan) was measured on the first and third leaf from the bottom of each plant to estimate leaf chlorophyll contents. Three measurements per leaf were taken at random positions on the leaf surface, and the mean value of three measurements was used for the analysis. In addition, leaf number of the whole shoot was counted for each plant at day 20.

Destructive sampling: At the day of sowing, eight seedlings of uniform growth from each genotype were sampled, oven-dried at 65 °C until constant weight. The whole seedlings were weighed. When the deepest root had reached the bottom of the rhizobox (at day 20), all plants were separately harvested and divided into above and below ground parts. The total leaf area was determined using a leaf area meter (LI-3100C, Licor, Lincoln, USA). The roots were carefully washed and the numbers of seminal and nodal roots were counted for each plant. Roots were scanned at a resolution of 600 dpi (STD 1600, Regent Instruments, Quebec, Canada), and root length and diameter distribution were determined using WinRHIZO software (version 2013, Regent Instruments, Quebec, Canada). We used root diameter as a criteria to distinguish between main roots and lateral roots according to [62]. Thus, roots with diameters >0.2 mm were considered as main roots, whilst <0.2 mm as lateral roots. The sum of all main roots and the sum of all lateral roots were used for further correlation and regression analyses. The shoots and roots were oven dried at 65 °C for 48 h to determine the root and shoot dry weights. Before drying, 3 cm-long nodal root samples were taken 3 cm from the root bases of every individual plant for anatomical measurements. The samples were preserved in 50% ethanol and stored at 4 °C in darkness until further analysis. Root cross sections of around 150 µm thickness were manually cut from each individual root sample with a razor blade and stained with Toluidine Blue (0.1% in distilled water) for 1 min. Cross sections were imaged at a resolution of 8 megapixel at 100× magnification using a digital microscope camera (Mirazoom MZ808, Oowl Tech Limited, Hong Kong, China), which was connected to a bright field microscope (Kern Optics OBF 122, Kern & Sohb GmbH, Balingen, Germany; Objective: 10× magnification, 0.25 numerical aperture). The cross-sectional areas of the root, the stele, the cortex, the aerenchyma, and the proportion of the cortex occupied with aerenchyma, as well as the radial diameter of 15 cortical cells were assessed in ImageJ version v1.52t (National Institute of Health, Bethesda, MD, USA).

Calculations of growth traits: We calculated relative growth rate (RGR) based on the means of the natural logarithm-transformed whole plant biomass values for each genotype [63]. Differences in RGR among genotypes were related to differences in leaf area ratio (LAR), leaf area productivity (LAP), plant N concentration (PNC), and plant N productivity (PNP) based on the following functional relationships among growth traits [27,64]:

RGR = LAR × LAP(2)

RGR = PNC × PNP(3)

Relative N accumulation rate reflects N accumulation pattern in a similar way as RGR does for the accumulation of biomass. The RGR, PNC, PNP and relative N accumulation rate were calculated with data from Day 1 and 20. The LAR and LAP were calculated with data from Day 7 and 20, because the leaf area was too small to conduct a reliable measurement before Day 7. However, all growth analysis calculations were done on a per-day basis and are therefore comparable.

In addition to the calculation of nutrient productivity described above for N, we also estimated the N and P productivities in a simplified procedure, because seedling P contents at the initial harvest could not be determined. Thus, assuming that the plants were grown under similar and constant (over time) conditions during a short period of time, we used the inverses of the final mass-based N and P concentrations as surrogates for the N and P productivities. In the case of N productivity, we aimed to verify the relationship between the two different estimates by means of correlation analysis.

### 4.3. Nutrient Analyses

At days 1 and 20, whole plants were sampled for nutrient analysis. Since the eight individual replicates were too small to allow for reliable element determinations, replicates were pooled to two batches prior to analysis. Oven-dried samples were ground in a stainless grinder to pass a 1 mm mesh before analysis. Nitrogen concentrations were analyzed for all plant samples from the initial and final harvests, using a LECO CN-2000 analyzer with a standard method (SS-ISO 13878). Plant samples only from the final harvest were digested in concentrated nitric acid on a heat block, and the concentrations of P, K, Ca, S, Mg, Mn, Fe, Cu, and Zn were determined using a Spectro Blue ICP analyzer using a standard method (SS 028311). We used the mean values of nutrient concentrations from the two batches for statistical analyses (means and standard errors are shown in Supplementary Table S4). The expected nutrient pools for achieving optimum nutrient ratios were calculated based on the suggested optimum N:P:K:Ca:Mg ratios of 100:14.3:68.3:8.3:8.7 by Knecht and Göransson [41], which have been explored previously for wheat [37].

### 4.4. Statistical Analyses

All statistical analyses were performed using R version 4.0.0 (R Core Team, 2020). One-way analysis of variance (ANOVA) was performed to analyze genotype effects on the traits measured at day 20. The leaf number, seminal root number and nodal root number were analyzed with generalized linear models (quasi-Poisson model with adjustment for under-dispersion) and Likelihood Ratio Chi-Square tests. The other traits were analyzed with linear models and F tests. Repeated measures ANOVA was performed to test genotype by time interaction effects for the following response variables: visible total root length, visible main root length, root system width and root system depth measured at days 4, 7, 11, 14, 18, and 20; total leaf area measured at days 7, 11, 14, and 18; and visible lateral root length at days 7, 11, 14, 18, and 20. Several linear-mixed models were tried, and the Akaike information criterion was used to choose the following model. Genotype, day and genotype by day interaction were treated as fixed effects. Block was treated as random effect. Observations from the same rhizobox were assumed to be correlated following an autoregressive covariance structure (corAR1 in the ‘lme’ function). The total leaf area, visible total root length, visible main root length and visible lateral root length were analyzed in log scales. The root system width and depth were analyzed in square root scales. A linear model and Tukey’s HSD test was used to analyze genotype differences in the slopes of linear relationships between total leaf area and total root length non-destructively measured along days 7, 11, 14, and 18. The relationships between biomass traits and nutrients, and the relationships between N and other nutrients were analyzed by linear regression and Pearson’s correlation. The relationship between whole plant biomass and N and P productivities (as the inverses of mass-based N and P concentrations) were analyzed by non-linear regression. The scaling relation between the stoichiometric niche volume of N and P on the one hand, and the volume of other nutrient elements on the other hand, were analyzed with reduced major axes (RMA) regression [42,65].

## Figures and Tables

**Table 1:** Analysis of variance (ANOVA) for genotype effect on various traits measured at day 20 for nine spring wheat genotypes.

Trait (Unit)	*p* Value	Description
**Non-destructive measurements**		
Leaf number (-)	<0.001 ***	Amount of leaves per plant
SPAD leaf1 (-)	<0.001 ***	Leaf chlorophyll content measured for the first leaf
SPAD leaf3 (-)	<0.001 ***	Leaf chlorophyll content measured for the third leaf
Visible total root length (cm)	<0.001 ***	Length of all visible roots in image
Visible main root length (cm)	<0.001 ***	Length of visible main roots in image
Visible lateral root length (cm)	0.021 *	Length of visible lateral roots in image
Visible root system depth (cm)	<0.001 ***	Maximal vertical depth of a visible root system in image
Visible root system width (cm)	0.009 **	Maximal horizontal width of a visible root system in image
**Measurements after destructive sampling**		
Shoot biomass (mg)	0.008 **	Dry weight of all shoots including leaves
Root biomass (mg)	0.031 *	Dry weight of all roots
Whole plant biomass (mg)	0.013 *	Sum of shoot and root biomass
Shoot fraction (%)	<0.001 ***	Shoot biomass per whole plant biomass
Total leaf area (cm^2^)	0.006 **	Total area of all leaves
Root fraction (%)	<0.001 ***	Root biomass per whole plant biomass
Total root length (m)	0.006 **	Total length of all roots
Total root surface area (m^2^)	0.019 *	Total area of all roots
Main root length (m)	0.179	Length of roots with diameter > 0.2 mm
Lateral root length (m)	<0.001 ***	Length of roots with diameter < 0.2 mm
Specific root length (m mg^−1^)	0.469	Total root length per root biomass
Total root length: total leaf area (m cm^−2^)	0.015 **	Root length per leaf area
Seminal root number (-)	<0.001 ***	Amount of seminal roots per plant
Nodal root number (-)	<0.001 ***	Amount of nodal roots per plant
Cross-sectional root area (µm^2^)	<0.001 ***	Cross-sectional area of a nodal root
Cross-sectional stele area (µm^2^)	0.001 **	Cross-sectional stele area of a nodal root
Cross-sectional cortex area (µm^2^)	<0.001 ***	Cross-sectional cortex area of a nodal root
Cross-sectional aerenchyma area (µm^2^)	0.642	Cross-sectional aerenchyma area of a nodal root
Aerenchyma proportion (%)	0.788	Cross-sectional aerenchyma area per cortex area of a nodal root
Cortical cell diameter (µm)	0.078	Mean diameter of 15 cortical cells in a nodal root

Note: One-way ANOVA was performed with eight replicates. The total leaf number, seminal root number and nodal root number were analyzed with generalized linear models and Likelihood Ratio Chi-Square tests. The other traits were analyzed with linear models and F tests (* significant at *p* ≤ 0.05, ** significant at *p* ≤ 0.01, *** significant at *p* ≤ 0.001).

**Table 2:** Pearson correlation coefficients and significant levels for various traits measured at day 20 for nine spring wheat genotypes.

	N	P	K	S	Ca	Mg	Shoot Biomass	Total Leaf Area	Total Root Length	Main Root Length	Lateral Root Length	Aerenchyma Proportion	Cortical Cell Diameter
**N**		0.102	<0.001 ***	0.003 **	0.001 ***	0.005 **	<0.001 ***	<0.001 ***	0.013 *	0.075	0.026 *	0.929	0.592
**P**	0.580		0.206	0.253	0.010 **	0.033 *	0.109	0.034 *	0.107	0.021 *	0.219	0.578	0.721
**K**	**0.944**	0.466		<0.001 ***	0.015 *	0.016 *	<0.001 ***	0.001 ***	0.009 **	0.070	0.018 *	0.906	0.641
**S**	**0.857**	0.426	**0.920**		0.036 *	0.054	0.004 **	0.016 *	<0.001 ***	0.126	<0.001 ***	0.658	0.188
**Ca**	**0.889**	**0.799**	**0.770**	**0.701**		<0.001 ***	<0.001 ***	<0.001 ***	0.023 *	0.070	0.045 *	0.611	0.679
**Mg**	**0.839**	**0.707**	**0.768**	0.658	**0.926**		0.005 **	0.004 **	0.035 *	0.029 *	0.080	0.804	0.896
**Shoot biomass**	**0.987**	0.569	**0.944**	**0.845**	**0.905**	**0.841**		<0.001 ***	0.018 *	0.116	0.029	0.890	0.666
**Total leaf area**	**0.900**	**0.706**	**0.902**	**0.766**	**0.901**	**0.847**	**0.935**		0.032 *	0.050 *	0.067	0.916	0.844
**Total root length**	**0.781**	0.573	**0.807**	**0.949**	**0.739**	**0.702**	**0.759**	**0.709**		0.067	<0.001 ***	0.413	0.127
**Main root length**	0.619	**0.747**	0.628	0.549	0.628	**0.718**	0.562	**0.667**	0.634		0.218	0.620	0.794
**Lateral root length**	**0.730**	0.455	**0.757**	**0.942**	**0.678**	0.611	**0.720**	0.633	**0.977**	0.456		0.270	0.086
**Aerenchyma proportion**	0.035	0.215	−0.046	0.172	0.197	0.097	0.054	−0.041	0.312	−0.192	0.412		0.729
**Cortical cell diameter**	0.207	0.139	0.181	0.482	0.161	−0.051	0.168	0.077	0.547	0.102	0.602	0.135	

Note: The values in the lower left corner refer to the Pearson correlation coefficients (*p* ≤ 0.05 in bold), and the values in the upper right corner are the corresponding p values (* significant at *p* ≤ 0.05, ** significant at *p* ≤ 0.01, *** significant at *p* ≤ 0.001). Nutrient element abbreviations represent the corresponding nutrient pools in shoots and roots at day 20. Root and shoot traits were from destructive sampling at day 20.

## Data Availability

The data supporting the finding of this manuscript are available on reasonable request to the corresponding author.

## Supplementary Materials

The following are available online at https://www.mdpi.com/2223-7747/10/1/174/s1, Table S1: Repeated measures ANOVA for genotype by time interactions regarding leaf and root traits measured along 20 days of growth. Table S2: Pearson correlation coefficients and significant levels for growth traits from nine spring wheat genotypes. Table S3: Pearson correlation coefficients and significant levels for nutrient accumulation and root traits from nine spring wheat genotypes. Table S4: Nutrient concentration in whole plants at day 20. Table S5: List of the nine spring wheat genotypes used in this study. Figure S1: Prediction of whole plant biomass at day 20 with nitrogen and phosphorus productivities. Figure S2: Relationship between the stoichiometric niche volume of nitrogen and phosphorus and the volume of other nutrient elements. Figure S3: Environmental conditions during the experiment.

## Abbreviations

LAP	leaf area productivity
LAR	leaf area ratio
PNC	plant nitrogen concentration
PNP	plant nitrogen productivity
RGR	relative growth rate of whole plant biomass
