# Supplementary material for: Shoot and Root Traits Underlying Genotypic Variation in Early Vigor and Nutrient Accumulation in Spring Wheat Grown in High-Latitude Light Conditions
Source: Plants (Basel). 2021 Jan 18;10(1):174. doi: 10.3390/plants10010174 (PMC7831908; doi:10.3390/plants10010174)
Supplement: Supplementary file 1 [file plants-10-00174-s001.pdf]

Article

Shoot and root traits underlying genotypic variation in early vigor and nutrient accumulation in spring wheat grown in high-latitude light conditions

Hui Liu [1,*], Fabio Fiorani [2], Ortrud Jäck [1], Tino Colombi [3], Kerstin A. Nagel [2] and Martin Weih [1]

[1] Department of Crop Production Ecology, Swedish University of Agricultural Sciences, 75007 Uppsala, Sweden
[2] Institute for Bio and Geosciences-2, Plant Sciences, Forschungszentrum Jülich GmbH, 52425 Jülich, Germany
[3] Department of Soil and Environment, Swedish University of Agricultural Sciences, 75007 Uppsala, Sweden

* Correspondence: hui.liu@slu.se

Supplementary Table S1. Repeated measures ANOVA for genotype by time interactions regarding leaf and root traits measured along 20 days of growth.

**Trait (Unit)**	***p* Value**
Total leaf area (cm^2)	0.012*
Visible total root length (cm)	< 0.001***
Visible Main root length (cm)	< 0.001***
Visible lateral root length (cm)	< 0.001***
Root system width (cm)	< 0.001***
Root system depth (cm)	< 0.001***

Linear-mixed models were used. Genotype, day and genotype by day interaction were treated as fixed effects. Block was treated as random effect. Total leaf area, visible total root length, visible main root length, and visible lateral root length were analyzed in log scales; root system width and root system depth were analyzed in square root scales (*indicates significant at $p \leq 0.05$, ** indicates significant at $p \leq 0.01$, *** indicates significant at $p \leq 0.001$).

Supplementary Table S2. Pearson correlation coefficients and significant levels for growth traits from nine spring wheat genotypes.

	**Relative Growth Rate**	**Leaf Area Ratio**	**Leaf Area Productivity**	**Nitrogen Productivity**	**Nitrogen Concentration**	**Total Leaf Area**	**Nodal Root Number**
**Relative growth rate**		0.178	0.036*	< 0.001***	0.456	0.031*	0.006**
**Leaf area ratio**	0.493		0.504	0.274	0.917	0.781	0.036*
**Leaf area productivity**	**0.699**	−0.258		0.050	0.475	< 0.001***	0.316
**Nitrogen productivity**	**0.907**	0.409	0.665		0.053	0.062	0.002**
**Nitrogen concentration**	−0.286	−0.041	−0.274	−0.661		0.654	0.166
**Total leaf area**	**0.713**	−0.109	**0.927**	0.643	−0.174		0.293
**Nodal root number**	**0.825**	**0.698**	0.378	**0.874**	−0.505	0.395	

The values in the lower left corner refer to the Pearson correlation coefficients ($p \leq 0.05$ in bold), and the values in the upper right corner are the corresponding *p* values (*significant at $p \leq 0.05$, ** significant at $p \leq 0.01$, *** significant at $p \leq 0.001$).

Supplementary Table S3. Pearson correlation coefficients and significant levels for nutrient accumulation and root traits from nine spring wheat genotypes.

	**K**	**S**	**Root System Width**	**Visible Lateral Root Length**
**K**		< 0.001***	0.127	0.030*
**S**	**0.920**		0.044*	0.007*
**Root system width**	0.574	**0.680**		0.293
**Visible lateral root length**	**0.717**	**0.816**	0.395	

The values in the lower left corner refer to the Pearson correlation coefficients ($p \leq 0.05$ in bold), and the values in the upper right corner are the corresponding p values (*significant at $p \leq 0.05$, ** significant at $p \leq 0.01$, *** significant at $p \leq 0.001$). The nutrient element abbreviations represent the corresponding nutrient pools in shoots and roots at day 20. The root system width was measured non-destructively at day 11, and visible lateral root length was measured non-destructively at day 14.

Supplementary Table S4. Nutrient concentration in whole plants at day 20 for nine spring wheat genotypes grown under controlled conditions in a phenotyping facility.

Genotype	N (mg/g)	Ca (mg/g)	K (mg/g)	Mg (mg/g)	P (mg/g)	S (mg/g)	Cu (mg/kg)	Fe (mg/kg)	Mn (mg/kg)	Zn (mg/kg)
KWS Alderon	54.58 ± 1.14	4.44 ± 0.07	65.73 ± 0.25	1.45 ± 0.01	6.27 ± 0.11	4.02 ± 0.10	9.78 ± 0.59	176.47 ± 12.48	39.27 ± 4.41	58.21 ± 3.15
Bjarne	55.90 ± 1.24	4.88 ± 0.09	62.77 ± 2.43	1.66 ± 0.08	6.80 ± 0.35	3.38 ± 0.02	10.77 ± 0.54	170.68 ± 8.45	32.01 ± 0.49	47.23 ± 2.39
Boett	54.28 ± 0.52	4.96 ± 0.13	55.27 ± 0.83	1.46 ± 0.03	7.37 ± 0.03	3.55 ± 0.01	9.77 ± 0.07	199.45 ± 0.16	37.37 ± 0.83	50.14 ± 4.67
Dacke	54.94 ± 0.43	5.07 ± 0.09	57.81 ± 1.51	1.79 ± 0.02	7.25 ± 0.20	3.37 ± 0.07	9.87 ± 0.27	212.80 ± 14.27	64.37 ± 7.60	55.10 ± 2.67
Dala landrace	53.90 ± 1.01	4.65 ± 0.08	61.79 ± 0.40	1.48 ± 0.03	6.56 ± 0.18	3.25 ± 0.02	7.97 ± 0.57	158.81 ± 6.53	29.88 ± 0.79	55.03 ± 1.44
Diskett	54.04 ± 0.47	4.78 ± 0.08	60.86 ± 1.01	1.53 ± 0.01	6.91 ± 0.12	3.51 ± 0.01	7.66 ± 0.44	181.40 ± 2.72	40.62 ± 5.95	46.07 ± 3.98
Happy	54.61 ± 2.19	4.75 ± 0.15	60.97 ± 0.37	1.54 ± 0.03	5.70 ± 0.01	3.46 ± 0.05	8.07 ± 0.27	162.70 ± 0.21	37.50 ± 0.86	46.67 ± 3.27
Quarna	56.29 ± 1.06	5.00 ± 0.03	56.99 ± 1.35	1.52 ± 0.01	7.43 ± 0.29	3.31 ± 0.12	10.79 ± 0.10	196.27 ± 33.26	50.22 ± 10.52	57.41 ± 5.00
Rohan	53.06 ± 0.50	5.14 ± 0.06	59.70 ± 1.80	1.66 ± 0.04	7.01 ± 0.26	3.51 ± 0.01	8.74 ± 0.04	238.27 ± 36.12	38.38 ± 0.59	44.79 ± 1.59

Each value represents the mean ± standard error (n = 2).

Supplementary Table S5. List of the nine spring wheat genotypes used in this study.

Genotype	Thousand Kernel Weight (g)	Accession No.	Origin
KWS Alderon	46	KWS W185	Germany
Bjarne	42.5	NK 97520	Sweden
Boett	44.5	SW 71034	Sweden
Dacke	39.5	W 26267	Sweden
Dala landrace	39.5	n/a (provided by local farmer)	Sweden
Diskett	43	SW 45456	Sweden
Happy	46.2	SW 91003	Sweden
Quarna	43	CH 21112283	Switzerland
Rohan	39.5	SW 01198	Sweden

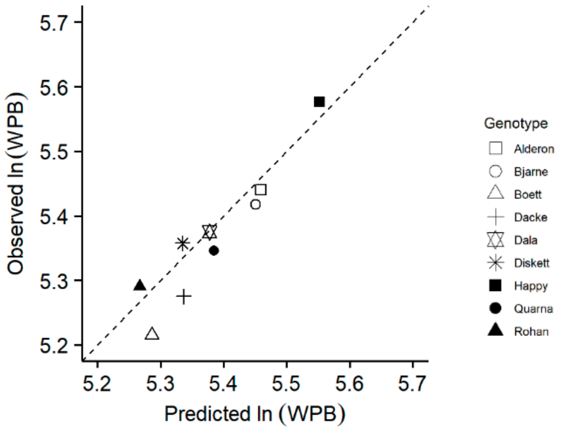

Supplementary Figure S1. Prediction of whole plant biomass (WPB) at day 20 with nitrogen (N) and phosphorus (P) productivities. Non-linear regression: ln (WPB) = 0.97 × ln (P productivity) − 2.96 × ln (N productivity) − 4.60; $r^2 = 0.698$, $p = 0.005$. The broken line indicates where the observed ln (WPB) equals the predicted value.

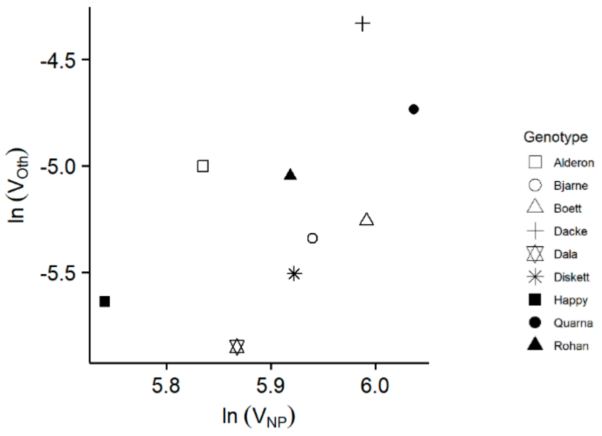

Supplementary Figure S2. Relationship between the stoichiometric niche volume of nitrogen and phosphorus and the volume of other nutrient elements. The volumes V_{NP} and V_{oth}, and the corresponding scaling exponent of -0.881 computed by reduced major axes regression, were calculated according to Ågren and Weih (2020), using the nutrient elements N and P on the one hand (V_{NP}) and K, Ca, S, Mg, Zn, Cu, Fe and Mn on the other hand (V_{Oth}).

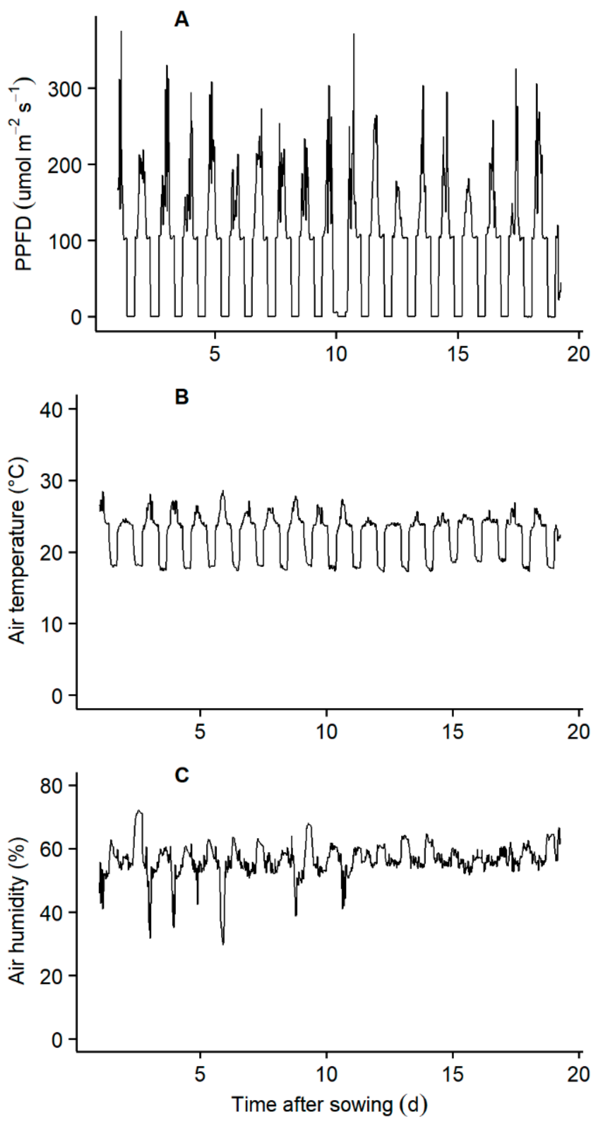

Supplementary Figure S3. Environmental conditions during the experiment. Eight sensors (one in each of the eight blocks) were used to monitor photosynthetic photon flux density (PPFD; A), air temperature (B) and air humidity (C) at plant level. Data were recoded every half an hour for 20 days. Means of data from eight sensors are shown in the figure.
